# Favourable humoral but reduced cellular immune response to COVID-19 mRNA BNT162b2 vaccine in patients with childhood-onset systemic lupus erythematosus

**DOI:** 10.1136/lupus-2024-001268

**Published:** 2024-09-20

**Authors:** Esra Karabag Yilmaz, Ayse Agbas, Nur Canpolat, Aybuke Gunalp, Sezgin Sahin, Dogukan Ozbey, Ruveyda Gulmez, Seha Kamil Saygili, Bekir Kocazeybek, Ozgur Kasapcopur, Salim Caliskan

**Affiliations:** 1 Department of Pediatric Nephrology, Istanbul University-Cerrahpasa, Cerrahpasa Faculty of Medicine, Istanbul, Turkey; 2 Department of Pediatric Rheumatology, Istanbul University-Cerrahpasa, Cerrahpasa Faculty of Medicine, Istanbul, Turkey; 3 Department of Microbiology, Istanbul University-Cerrahpasa, Cerrahpasa Faculty of Medicine, Istanbul, Turkey

**Keywords:** COVID-19, Lupus Erythematosus, Systemic, Vaccination

## Abstract

**Objective:**

To evaluate both humoral and cellular immune responses to the COVID-19 messenger RNA (mRNA; BNT162b2) vaccine in patients with childhood-onset SLE (cSLE) compared with healthy controls and patient controls (kidney transplant (KTx) recipients).

**Methods:**

This single-centre, cross-sectional and case–control study included 16 patients with cSLE, 19 healthy controls and 19 KTx recipients. We assessed SARS-CoV-2-specific humoral (anti-SARS-CoV-2 IgG, neutralising antibody (nAb)) and cellular (interferon gamma release assay (IGRA)) immune responses at least 1 month after administration of two doses of the mRNA vaccine.

**Results:**

Humoral immune response rates (anti-SARS-CoV-2 IgG and nAb seropositivity) in patients with cSLE were comparable to healthy controls (100% vs 100% and 100% vs 95%, respectively) but significantly higher than in KTx recipients (74% and 42%, p<0.05 for both). Cellular immune response rate measured by IGRA was lower in patients with cSLE compared with healthy controls (56.3% vs 89.5%, p=0.050) and comparable to KTx recipients (63%). IGRA-negative patients with cSLE had significantly lower total leucocyte and lymphocyte counts at vaccination time as compared with their counterparts (p=0.008 and p=0.001, respectively). No differences were found in disease activity or immunosuppressive therapies between IGRA-negative and IGRA-positive patients with cSLE.

**Conclusion:**

Patients with cSLE showed robust humoral but compromised cellular immune responses to the COVID-19 mRNA vaccine, associated with lower lymphocyte counts. These findings highlight the need for further research to enhance vaccine efficacy in this vulnerable group.

**WHAT IS ALREADY KNOWN ON THIS TOPIC**

- Research on adult patients with SLE has demonstrated a favourable humoral but diminished cellular immune response to COVID-19 messenger RNA (mRNA) vaccines.
- Additionally, reduced immunogenicity to mRNA vaccines has been linked to immunosuppressive treatment, particularly with mycophenolate mofetil (MMF).
- Data are scarce for childhood-onset SLE (cSLE).

**WHAT THIS STUDY ADDS**

- This study evaluates the humoral and cellular immune responses to COVID-19 mRNA vaccines in patients with cSLE, comparing them with healthy individuals and kidney transplant (KTx) recipients.
- The findings reveal that the humoral immune response in patients with cSLE is strong, comparable to that of healthy controls and superior to that of KTx recipients after two doses of the vaccine.
- Conversely, the cellular immune response in patients with cSLE is weaker than that of healthy controls and as low as KTx recipients.
- Notably, a low lymphocyte count, rather than immunosuppressive treatment, particularly MMF, appears to be associated with this reduced cellular immune response.

**HOW THIS STUDY MIGHT AFFECT RESEARCH, PRACTICE OR POLICY**

- The study highlights the need for further studies involving larger cohorts to confirm these findings and enhance vaccine responses.
- Such studies could significantly influence clinical practices and policies by identifying strategies to optimise vaccine efficacy in these vulnerable patients.

## Introduction

 Patients with SLE may face challenges with immune regulation, potentially leading to diminished immune responses to vaccines due to active disease or immunosuppressive therapy.^1^ It is considered that individuals with SLE may have reduced humoral or cellular responses to the SARS-CoV-2 messenger RNA (mRNA) vaccine than healthy individuals.^2^,^6^ While studies in adults generally have shown a favourable humoral immune response to the mRNA vaccine, reduced immunity appears to be linked to the use of immunosuppressive therapies, particularly mycophenolate mofetil (MMF).^5^,^8^ Limited research on cellular immune response has shown reduction in those with poor humoral immune responses.^6 7^

Similarly, evidence regarding the immune response to COVID-19 vaccination in childhood-onset SLE (cSLE) mirrors that of adults, with generally favourable humoral immune responses.^9^,^13^ MMF has been implicated as a main factor contributing to diminished humoral immune response,^9 12^ although this effect is not consistently observed following the second dose of vaccination.^14^ All these studies,^9^,^13^ except one,^14^ have been assessed alongside individuals with various rheumatic diseases. Cellular immune response has been specifically evaluated in only one paediatric study to date, which showed favourable humoral and cellular immune responses among individuals receiving both high and low immunosuppressive treatments.^14^

Given the limited data available for the cSLE population, this study aims to evaluate both humoral and cellular immune responses to SARS-CoV-2 vaccination in this patient population. Moreover, we aimed to compare these immune responses with those of healthy controls and also with kidney transplant (KTx) recipients, another paediatric cohort receiving MMF, to serve as a patient control, since MMF is implicated in reducing immune responses in patients with SLE.

## Materials and methods

### Study design and study population

This single-centre, cross-sectional and case–control study was conducted at the Department of Paediatric Nephrology and Department of Paediatric Rheumatology, Istanbul University-Cerrahpasa, Cerrahpasa Medical Faculty, between April 2022 and September 2022. Written informed consent was obtained from parents and patients over 10 years of age.

A total of 59 actively followed patients with cSLE, fulfilling the 2012 Systemic Lupus Erythematosus International Collaborating Clinics (SLICC) criteria,^15^ aged 12–21 years and receiving immunosuppressive treatment were included. Patients were asked about receipt of the BNT162b2 mRNA COVID-19 vaccine (Pfizer-BioNTech). Two doses of the vaccine were recommended for patients who had not been vaccinated or who had only received the first dose. Ultimately, those who completed two doses of the vaccine were included in this study. The following exclusion criteria were applied to all participants: receipt of a non-BNT162b2 vaccine, incomplete administration of two doses of the BNT162b2 vaccine, vaccine hesitancy, primary immunodeficiency and refusal to participate. Six teen patients with cSLE were enrolled in the study (patient group), none of whom received a third dose during the study period. Their clinical characteristics (disease and treatment features) were recorded from their medical records. Disease flare was defined as a Systemic Lupus Erythematosus Disease Activity Index 2000 update (SLEDAI-2K) score ≥4.^16^ COVID-19 infection history was noted, and SARS-CoV-2 IgG nucleocapsid protein antibody (NCP Ab) testing was performed to discover prior wild-type SARS-CoV-2 infection.

To compare the vaccine response in patients with cSLE, we used two control groups: a ‘healthy control group’ of 19 age-comparable and sex-comparable children and a ‘patient control group’ consisting of 19 paediatric KTx recipients who received two doses of the BNT162b2 vaccine. None of them had a third dose vaccine during the study period. These control groups were selected from our previously published multicentre study.^17^ Specifically, KTx recipients aged between 12 and 21 years and exclusively followed at our centre (Department of Paediatric Nephrology, Cerrahpasa Medical Faculty) were selected as patient controls. Notably, the humoral and cellular immune responses were assessed in the same laboratory using the same methodology and kits in both the previous and the current studies.

### Assessment of immune response to SARS-CoV-2 vaccine

To analyse the humoral and cellular immune response to the vaccine, serum and whole blood samples were collected from all patients and controls at least 1 month after the second vaccine dose. All samples were stored at −20°C until testing.

#### Humoral response

Anti-SARS-CoV-2 IgG quantification (anti-SARS-CoV-2 IgG) and neutralisation test (neutralising antibody (nAb) activity) were used to assess humoral immune response. Anti-SARS-CoV-2 IgG antibody titres were determined by SARS-CoV-2 QuantiVac ELISA (IgG) (Euroimmun AG, Lübeck, Germany). The neutralisation capacities of these antibodies were also determined by SARS-CoV-2 NeutraLISA (Euroimmun AG). Antibody titres were expressed in relative units per millilitre (RU/mL) (1 RU/mL×3.2=1 binding antibody unit per millilitre (BAU/mL)). Antibody titre values below 8 RU/mL (25.6 BAU/mL) were interpreted as ‘seronegative’ and those above 11 RU/mL (35.2 BAU/mL) were interpreted as ‘seropositive’, according to the manufacturer’s guidelines. Serum samples that exceeded the assay measuring range (>120 RU/mL) were diluted by a 1:10 factor and retested to obtain more accurate results. nAb responses were assessed as per cent inhibition (%IH). %IH values below 20% were considered ‘nAb-negative’ and those above 35% were considered ‘nAb-positive’, according to the manufacturer’s guidelines.

#### Cellular immune response

Interferon gamma release assay (IGRA) test was used to assess cellular immune response. A specific stimulation of T cells by the spike protein of SARS-CoV-2 was performed using the Quan-T-Cell SARS-CoV-2 (Euroimmun AG) to determine the amount of the interferon gamma released by immune cells. The interferon gamma responses were then measured using the Quan-T-Cell ELISA (Euroimmun AG). The results were expressed in mIU/mL in accordance with the manufacturer’s instructions. Values below 100 mIU/mL were interpreted as ‘IGRA-negative’ and values above 200 mIU/mL were interpreted as ‘IGRA-positive’.

#### SARS-CoV-2 IgG NCP Ab test

The SARS-CoV-2 IgG test (ARCHITECT IgG test, Abbott, USA), which semiquantitatively detects IgG antibodies against the NCP of SARS-CoV-2 using the chemiluminescent microparticle immunoassay method, was used. The results obtained from all sera studied were given as index specimen/calibrator (S/C) units (the strength of response in relative light units reflects the quantity of IgG present and is compared with a calibrator to determine the calculated index S/C for a sample) and were evaluated as <1.4 S/C negative and ≥1.4 S/C positive.^18^

### Statistical analyses

The SPSS (Statistical Package for Social Sciences) for Windows V.20.0 was used for analysis. Continuous data were expressed as median (25th and 75th percentiles) and analysed using the Mann-Whitney U test or the Kruskal-Wallis test. Categorical variables were expressed as n (%) and analysed with χ^2^ test. Logarithmic transformation was applied to the titres of anti-SARS-CoV-2 IgG, IGRA and nAb activity due to non-normally distributed data. The non-parametric Spearman rank-order correlation was used for non-normally distributed data. Variables with a p value of <0.05 were defined as significant.

## Results

### Study population and clinical characteristics

The median (IQR) age of patients with cSLE was 17.1 (13.9, 18.8) years, with a median follow-up duration of 63.8 (43.5, 164.3) months. Table 1 provides a detailed overview of the clinical characteristics. During the study period, none of the patients with cSLE experienced disease flare postvaccination. The median SLEDAI-2K score remained stable before and after vaccination as well as at the time of immune evaluation (2 (0, 4.0) vs 2 (0, 4.75), p=0.65). The median levels of C3 and C4 were 0.91 (0.72, 1.1) g/L and 0.14 (0.09, 0.16) g/L, respectively. The median anti-double-stranded DNA (IgG) was measured at 10 (3.7, 258) IU/mL. Two patients exhibited significant proteinuria that did not reach nephrotic range (urine protein/creatinine ratio ranged between 0.2 mg/mg and 2 mg/mg) at the time of vaccination; their proteinuria did not worsen postvaccination.

**Table 1:** Characteristics of patients with cSLE in comparison with healthy and patient control groups

	Patients with cSLE, n=16	Patient controls (KTx) n=19	Healthy controlsn=19	P value
Clinical characteristics				
Age, years	17.1 (13.9, 18.8)	17.1 (15.5, 21)	17.7 (13.8, 18.6)	0.27
Sex (female), n (%)	12 (75)	8 (42)	10 (53)	0.18
Time after second dose of vaccination, weeks (sampling time)	27.7 (9.4, 28.1)^a^	6.5 (4.9, 12)^b^	18.1 (5.2, 27.8)^a,b^	**0.001**
History of COVID-19 infection, n (%)	9 (56)^a,b^*	4 (21)^b^	11 (61)^b^	**0.03**
Age at the start of IS treatment, years	11.8 (0.8, 15.2)	10.7 (9, 13)		0.48
Time from the start of IS treatment to vaccination, years	3.9 (2.5, 8.4)	5.9 (2.9, 8.3)		0.65
Follow-up duration, months	63.8 (43.5, 164.3)	87 (55, 121)		0.83
Laboratory findings				
V-white cell count, ×10^9^/L	5.6 (5.5, 6.8)	9 (7, 10.3)	NA	**<0.001**
V-lymphocytes, ×10^9^/L	1.9 (1.4, 2.2)	2.6 (2.1, 3.4)	NA	**0.003**
V-neutrophils, ×10^9^/L	4.3 (3.6, 4.8)	5.2 (4.5, 6.8)		**0.011**
eGFR, mL/min/1.73 m^2^	118 (108.5, 134.7)	64 (54.8, 81)		**<0.001**
Immunosuppression treatment				
Multiple immunosuppressive agents, n (%)†	5 (31)	19 (100)		**<0.001**
Steroid use, n (%)	10 (62)	19 (100)		**0.004**
Steroid dose >10 mg/day, n (%)	5 (50)	0		**0.002**
MMF, n (%)	6 (37.5)	19 (100)		**<0.001**
MMF dose, mg/m^2^	1166 (714, 1333)	680 (651, 757)		0.07
AZA, n (%)	5 (31)	0		**0.013**
CNI, n (%)	0	19 (100)		**<0.001**
HCQ, n (%)	15 (94)	0		**<0.001**
CYC, n (%)‡	1 (6)	0		0.46
RTX, n (%)‡	0	0		

Data are presented as median (25th,; 75th percentile) or n/Nn (%). Continuous data were analysed by the Mann–-Whitney U test for two -group and the Kruskal–-Wallis test for three -group comparisons. χ2Chi-square test or Fisher’sFischers Eexact test werewas used for categorical variables, where appropriate. Different letters superscripts (a,b) within a row represents significant differences between medians at the level after corrections for multiple comparisons. P values lower than 0.05 are given in bold.

Different superscript letters (a, b) within a row represent significant differences between medians at the 0.05 alpha level after corrections for multiple comparisons.

P values lower than 0.05 are given in bold.

* Nucleocapsid protein antibody test indicates prior COVID-19 infection was assessed only in patients with cSLE.

† Patients with cSLE were receiving steroid and MMF or AZA; KTx recipients were receiving steroid, MMF and CNI.

‡ Receiving 6 months prior to the vaccination.

AZA, azathioprine; CNI, calcineurin inhibitors; cSLE, childhood-onset SLE; CYC, cyclophosphamide; eGFR, estimated glomerular filtration rate; HCQhydroxychloroquineISimmunosuppressiveKTx, kidney transplant recipients; MMF, mycophenolate mofetil; NA, not available; RTX, rituximab; V, white cell count, lymphocytes and neutrophil count at vaccination

Comparative analysis with two groups, shown in table 1, revealed no significant differences in age and sex distribution among the three groups. The interval since the second vaccine dose was longer in patients with cSLE compared with both KTx recipients and healthy controls. However, the difference was only statistically significant between patients with cSLE and KTx recipients (p=0.001). In addition, at the time of the first vaccination, total leucocyte and lymphocyte counts were significantly lower in patients with cSLE compared with KTx recipients (p<0.001 and p=0.003, respectively).

### Immunosuppressive therapy

The median age at the initiation of immunosuppressive therapy for patients with cSLE was 11.8 (0.8, 15.2) years, which was comparable to that of KTx recipients, at 10.7 (9, 13) years (p=0.48).

Among patients with cSLE, 67% (n=11) were receiving more than one immunosuppressive agent. A high proportion (94%, n=15) were on hydroxychloroquine, while 37.5% (n=6) were on MMF and 31% (n=5) were on azathioprine. Additionally, 62% (n=10) were receiving corticosteroid (CS) with a median dose of 0.2 (0.08, 0.75) mg/kg, of which five were on doses exceeding 10 mg/day. None of the patients with cSLE had a history of receiving other immunosuppressants such as calcineurin inhibitors (CNI), methotrexate or rituximab, except one who received cyclophosphamide 6 months prior to vaccination (table 1). The regimen of immunosuppressive treatments of patients with cSLE remained unchanged at the time of sampling postvaccination.

Conversely, all KTx recipients, serving as a patient control group, were on triple immunosuppressive therapy, including CS, MMF and CNI. Of the KTx recipients, 10 (52%) received antithymocyte globulin and 7 (37%) received basiliximab as induction therapy, all administered at least 1 year before vaccination. During follow-up, none of the KTx recipients experienced cellular rejection, but one patient had antibody-mediated rejection and received rituximab 1 year before vaccination.

As shown in table 1, patients with cSLE had significantly lower use of multiple immunosuppressive therapy (p<0.001), MMF (p<0.001) and CS (p=0.004) compared with KTx recipients. However, patients with cSLE were receiving a higher dose of MMF than KTx recipients, with borderline significance (p=0.07). In addition, 31% of patients with cSLE but none of KTx recipients were on CS doses exceeding 10 mg/day.

### Humoral immune response

**Figure 1:**
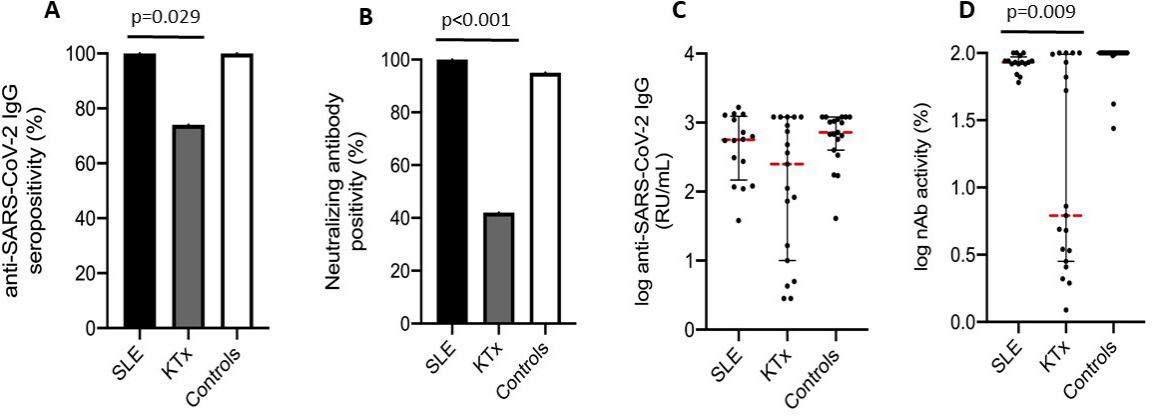
Comparison of humoral immune responses between patients with SLE, patient controls (KTx recipients) and healthy controls. (**A**) Anti-SARS-CoV-2 IgG seropositivity rate, (**B**) nAb positivity rate, (**C**) anti-SARS-CoV-2 IgG titre and (D) nAb activity. Only differences between groups with p<0.1 are shown. KTx, kidney transplant; nAb, neutralising antibody; RU/mL, relative units per millilitre.

Anti-SARS-CoV-2 IgG positivity was 100%, 74% and 100% in patients with cSLE, KTx recipients and healthy controls, respectively. Anti-SARS-CoV-2 IgG positivity in patients with cSLE was comparable to healthy controls but significantly higher than in KTx recipients (p=0.029) (figure 1A). Similarly, nAb positivity in patients with cSLE was comparable to healthy controls (100% vs 95%, p=1.00), but significantly higher than in KTx recipients (100% vs 42%, p<0.001) (figure 1B).

The median anti-SARS-CoV-2 IgG titres of patients with cSLE (564 (159, 1239) RU/mL) did not differ from those of KTx recipients (252 (10, 1200) RU/mL) or healthy controls (731 (394, 1200) RU/mL) (figure 1C). However, the median nAb activity of patients with cSLE was lower than that of healthy controls, with borderline statistical significance (85.7% (83.5%, 93%) vs 99.4% (98.8%, 99.5%) p=0.07), but significantly higher than in KTx recipients (85.7% (83.5%, 93%) vs 6.1% (2.8%, 98.6%) p=0.009) (figure 1D).

We found no correlation between sampling time (post-second vaccination) and anti-SARS-CoV-2 IgG or nAb activity in patients with cSLE, KTx recipients or healthy controls.

### Cellular immune response

**Figure 2:**
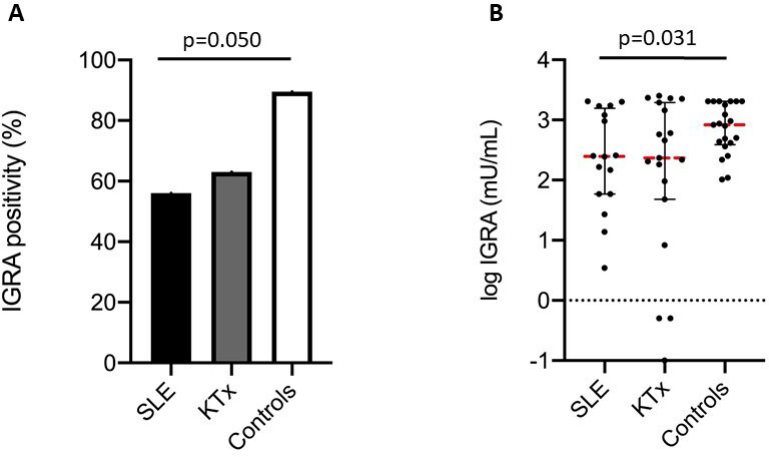
Comparison of cellular immune responses between patients with SLE, patient controls (KTx recipients) and healthy controls. (**A**) IGRA positivity rate and (**B**) IGRA titre. Only differences between groups with p<0.1 are shown. IGRA, interferon gamma release assay; KTx, kidney transplant.

IGRA positivity was found in 56.3% of patients with cSLE, 63% of KTx recipients and 89.5% of healthy controls (figure 2A). Patients with cSLE had significantly lower IGRA positivity than healthy controls (p=0.050) but were similar to KTx recipients (p=0.68). Patients with cSLE also had significantly lower IGRA titres than healthy controls (247 (58.7, 1581) mIU/mL vs 841 (407, 2057) mIU/mL, p=0.031), but similar to KTx recipients (236 (47.5, 1947) mIU/mL, p=0.88) (figure 2B).

We found no correlation between sampling time (post-second vaccination) and IGRA titres in patients with cSLE, KTx recipients or healthy controls.

### Effect of prior COVID-19 infection on vaccine response

Out of 16 cSLE patients, 9 (56.3%) had NCP antibodies, indicating prior COVID-19 infection. There were no differences in anti-SARS-CoV-2 IgG (100% vs 100%), nAb (100% vs 100%) and IGRA positivity (67% vs 43%, p=0.35), as well as in anti-SARS-CoV-2 IgG titres (626 (411, 1314) vs 306 (109, 1095), p=0.21), nAb activity (85.8% (83.7%, 99.4%) vs 85.6% (83.7%, 99.4%), p=0.56) or IGRA titres (249 (102.4, 1727) vs 164 (27, 12 000), p=0.68), between patients with cSLE with and without NCP antibody positivity. Four out of 19 KTx recipients (21%) and 11 out of 19 healthy controls (61%) had a history of COVID-19 infection. There were also no differences in anti-SARS-CoV-2 IgG, nAb or IGRA positivity or titres between patients with and without history of COVID-19 infection within each group.

### Factors affecting cellular immunity in patients with SLE

As shown in table 2, no differences were found between IGRA-negative (n=7) and IGRA-positive (n=9) patients with cSLE in disease duration, SLEDAI-2K score, complement levels, positivity for any SLE antibodies, CS use or CS >10 mg/day, MMF use or daily dose, and estimated glomerular filtration rate. However, the total leucocyte and lymphocyte counts at vaccination time were significantly lower in the IGRA-negative patients with cSLE (p=0.008 and p=0.001, respectively).

**Table 2:** Factors affecting reduced cellular response in patients with cSLE

	IGRA-positiven=9	IGRA-negativen=7	P value
Clinical characteristics			
Age, years	16.7 (16.3, 17.1)	13.7 (13.5, 13.9)	0.68
Sex (female), n (%)	8 (89)	4 (57)	0.15
Age of onset of disease, years	12.3 (11.8, 12.8)	5.3 (0.8, 9.9)	0.07
Follow-up duration, months	52.8 (41.9, 63.8)	106.4 (48.6, 164.3)	0.30
V-SLEDAI-2K score	4.0 (0.0, 4.0)	1.0 (0.0, 2.0)	0.40
S-SLEDAI-2K score	2.0 (0.0, 4.0)	2.0 (0.5, 3.5)	0.83
Time after second dose of vaccination, weeks	27.9 (27.7, 28.1)	21.3 (9, 33.6)	0.95
Laboratory findings			
eGFR (mL/min/1.73 m^2^)	98.8 (73, 125)	102.5 (97, 108)	0.60
V-white cell count, ×10^9^/L	8.3 (6.7, 9.9)	5.6 (5.4, 5.8)	**0.008**
V-lymphocytes, ×10^9^ /L	2.5 (2.0, 3.0)	1.05 (0.5, 1.6)	**0.001**
V-neutrophils, ×10^9^ /L	4.4 (3.7, 5.9)	4.2 (1.4, 4.8)	0.42
S-lymphocytes, ×10^9^ /L	1.6 (1.3, 1.9)	1.95 (0.30, 3.6)	0.17
V-C3 (0.9–1.8 g/L)	0.86 (0.67, 1.0)	0.98 (0.91, 1.0)	0.53
V-C4 (0.1–0.4 g/L)	0.12 (0.09, 0.15)	0.36 (0.05, 0.68)	0.43
V-dsDNA (IgG) (<12 IU/mL)	154.7 (9.5, 300)	129.5 (1.1, 258)	0.64
S-C3 (0.9–1.8 g/L)	0.81 (0.59, 1.0)	1.05 (1.0, 1.1)	0.95
S-C4 (0.1–0.4 g/L)	0.11 (0.06, 0.16)	0.38 (0.11, 0.65)	0.53
S-dsDNA (IgG) (<12 IU/mL)	149.4 (8.8, 290.0)	26.4 (0.85, 52.0)	0.91
SLE antibody positivity, n (%)			
Anti-smooth muscle antibodies	1/9 (11)	1/6 (17)	0.65
Anti-SS-A	1/9 (11)	2/6 (33)	0.52
Anti-SS-B	0	2 (33)	0.07
Anti-cardiolipin antibodies	3/9 (33)	2/7 (29)	0.84
Anti-β2-glycoprotein 1	1/9 (11)	2/7 (29)	0.55
Anti-phospholipid antibodies	0	2/7 (29)	0.10
Lupus anticoagulants	0	4/7 (57)	0.50
Immunosuppressive treatment			
Steroid use, n (%)	4/9 (44)	6/7 (86)	0.14
Steroid dose, mg/kg	0.08 (0.08, 0.14)	0.6 (0.18, 1.2)	0.19
Steroid dose >10 mg/day, n (%)	1/4 (25)	4/6 (67)	0.52
MMF/AZA, n (%)	5/9 (57)	6/7 (86)	0.30
MMF, n (%)	3/9 (33)	3/7 (43)	0.70
MMF dose, mg/m²	824 (316, 1333)	1000 (857, 1166)	1.00
AZA, n (%)	2/9 (22)	3/9 (43)	0.39
HCQ, n (%)	8/9 (89)	7/7 (100)	0.56
HCQ dose, mg/kg	3.5 (3, 4)	6 (5.5, 6)	0.07

Continuous data are presented as median (25th,; 75th percentile) and compared with Mann–-Whitney U test. Categorical data are given as n/nn (%) and compared with χ2Chi-square test. P values lower than 0.05 are given in bold

P values lower than 0.05 are given in bold.

anti-SS-A, anti-Sjögren’s syndrome-related antigen A; anti-SS-B, anti-Sjögren’s syndrome-related antigen B; AZA, azathioprine; cSLE, childhood-onset SLE; dsDNA, double-stranded DNA; eGFR, estimated glomerular filtration rate; HCQ, hydroxychloroquineIGRA, interferon gamma release assay; MMF, mycophenolate mofetil; S, sampling time; SLEDAI-2K, Systemic Lupus Erythematosus Disease Activity Index 2000; V, vaccination time

## Discussion

In this single-centre study, we evaluated both humoral and cellular immune responses to two doses of BNT162b2 mRNA COVID-19 vaccine in patients with cSLE. The present study revealed that patients with cSLE showed a strong humoral immune response but a reduced cellular immune response to the mRNA COVID-19 vaccine. Our study also revealed that the cellular immune response in patients with cSLE was as low as in KTx recipients, who received more immunosuppressive therapy. Furthermore, we observed that the reduced cellular immune response in patients with cSLE was associated with a low lymphocyte count at vaccination time but showed no clear association with disease activity or MMF use.

Studies have shown that immune responses decrease approximately 6–12 weeks after vaccination.^6 8 14^ Our assessment of humoral and cellular immunity post-mRNA vaccination occurred later than in previous studies, which might explain the observed reduction in cellular immune response. Despite this, the timing of sampling was consistent between patients with cSLE and healthy controls. Additionally, the humoral immune response in patients with cSLE was as favourable as that in healthy controls.

The prevalence of humoral immune responses to SARS-CoV-2 mRNA vaccines in adult patients with SLE varies widely due to differences in study protocols, established cut-off values and assay sensitivities. A systematic review reported a pooled seroconversion rate of 91.3% following administration of the mRNA vaccine in adult patients with SLE.^4^ The use of immunosuppressive agents such as glucocorticoids, methotrexate and especially MMF has been linked to diminished humoral immune response.^5^,^8^ In addition, a lower number of naïve B cells at the time of vaccination has also been related to poor vaccine response, although this relationship has not been established with the total lymphocyte count.^7^

In children and adolescents with SLE, vaccine responses have mostly been evaluated in different patient groups with rheumatic diseases and the seropositivity rate has ranged between 91% and 97.3%.^9 12^ Heshin-Bekenstein *et al*^9^ included 37 patients with autoimmune inflammatory rheumatic diseases (AIIRDs) and found a seropositivity rate of 97.3% in all patients and 100% in healthy controls. In this cohort, the mean anti-spike 1/spike 2 antibody titre of 10 patients with SLE was 229.5 BAU/mL, while the controls had a titre of 387 BAU/mL. Another study reported a seropositivity rate of 89% in 8 patients with cSLE within a cohort of 124 patients with AIIRDs.^12^ In that study, the mean anti-spike 1/spike 2 antibody titre of patients with cSLE was 152.77 BAU/mL, while the controls had a titre of 212.93 BAU/mL with 100% seropositivity. The authors noted that MMF therapy was associated with lower antibody levels, which improved after the third dose. Piyaphanee *et al*^14^ reported a seropositivity rate of 97.3% in adolescent SLE (adoSLE) patients. Individuals on high immunosuppressive therapy (>7.5 mg/day of prednisolone or taking another immunosuppressant) had lower anti-receptor-binding-domain IgG levels 4 weeks after the second dose of vaccine compared with those on low immunosuppressive therapy (≤7.5 mg/day of prednisolone and no immunosuppressant) (551.2 (95% CI 289.6 to 1048.9) BAU/mL vs 3684.1 (95% CI 2911.3 to 4661.9) BAU/mL). In our study, all patients with cSLE were seropositive for anti-SARS-CoV-2 IgG and seropositivity rate was higher than those reported in previous studies of adults and adoSLE patients.^4 14^ Also, the antibody titres of the patients were also comparable to those of healthy controls. In addition, patients with cSLE exhibited higher seropositivity and titres of anti-SARS-CoV-2 IgG positivity than KTx recipients, who were characterised by more intensive immunosuppressive medication.

nAb activity and positivity represent the clinical effectiveness of vaccine-induced antibodies. Limited studies in adult patients with SLE have reported nAb positivity rates to SARS-CoV-2 mRNA vaccines ranging between 42% and 92%.^5 8^ In the study with a 92% positivity rate, nAb activity was significantly lower than in healthy controls, and low nAb activity levels were associated with CS dose and MMF.^5^ The nAb positivity rate among patients with cSLE was reported at 44% in one paediatric study by Yeo *et al*.^13^ In this study, it was observed that the use of MMF in patients with cSLE reduced nAb positivity after the first dose of vaccination, but this effect was no longer present after the second dose of vaccination. The other paediatric study by Piyaphanee *et al*^14^ showed that adoSLE on high immunosuppressives had lower nAb levels than those on low immunosuppressives. In contrast to previous adult and paediatric studies, the nAb positivity rate in our cohort was 100%. However, the nAb activity in patients with cSLE was lower than in healthy controls, with borderline significance. Consequently, the seropositivity and nAb response of our paediatric cohort were better than previous data and as favourable as that of healthy controls. This is consistent with the humoral response of healthy adolescents, which has been reported to be better than that of healthy adults.^19^

Limited data are available in cellular immune response for patients with SLE. Reported cellular immune response rates in adult patients with SLE varied between 36% and 53%, while rates in paediatric patients ranged from 68% to 94%.^4 6 7 14^ These studies have not identified clear associations with low cellular immune response but have shown a positive correlation between humoral (anti-SARS-CoV-2 Ab and nAb) and cellular (IGRA titres) immune responses.^6 7^ In a study evaluating adoSLE, where cellular immunity was measured using interferon gamma ELISA (ELISpot), the high immunosuppressive group had a lower cellular immune response than the low immunosuppressive group.^14^ In our cohort of patients with cSLE, the cellular immune response (IGRA positivity) was 56%, consistent with adult studies, but significantly lower than healthy controls. Although patients with cSLE received less intense immunosuppressive therapy than KTx recipients, their cellular immune response was as low as KTx (56% vs 63%). It is also important to note that neither the use nor the dose of MMF differed between IGRA-positive and IGRA-negative patients with cSLE. Therefore, low cellular immune response could not have linked to MMF use alone. On the other hand, IGRA-negative patients had lower lymphocyte count at the time of vaccination, although disease activity did not significantly differ between the IGRA-positive and IGRA-negative patients with cSLE. Thus, patients with cSLE exhibited poor cellular immune response linked to low leucocyte counts. However, it remains challenging to determine whether low lymphocyte counts were due to disease activity or MMF dose. Further studies with larger numbers of patients are required to provide a more comprehensive insight.

We acknowledge several limitations to our study, including its cross-sectional design and the small cohort size. Due to the cross-sectional nature of the study, the sampling occurred later than 4 weeks. In addition, we included patients with cSLE with prior COVID-19 infection and analysed NCP antibodies to evaluate vaccine response based on NCP antibody positivity. However, we were unable to perform NCP antibody tests for the historical controls, KTx and healthy control groups. Despite these limitations, a key strength of our single-centre study is its comprehensive evaluation of both humoral and cellular immune responses to the BNT162b2 mRNA COVID-19 vaccine in patients with cSLE, specifically compared with healthy and patient controls receiving immunosuppressives.

In conclusion, this study provides valuable insights into the immune response of patients with cSLE to the BNT162b2 mRNA COVID-19 vaccine. While the humoral immune response is robust, the cellular immune response is compromised, potentially influenced by low lymphocyte count. Further studies with larger cohorts are warranted to confirm these findings and optimise vaccine responses.

## Data Availability

Data are available upon reasonable request.
